# Responsive Polydiacetylene Vesicles for Biosensing Microorganisms

**DOI:** 10.3390/s18020599

**Published:** 2018-02-15

**Authors:** Estelle Lebègue, Carole Farre, Catherine Jose, Joelle Saulnier, Florence Lagarde, Yves Chevalier, Carole Chaix, Nicole Jaffrezic-Renault

**Affiliations:** 1 Institute of Analytical Sciences, University of Lyon, 69100 Villeurbanne, France; carole.farre@isa-lyon.fr (C.F.); catherine.jose@isa-lyon.fr (C.J.); joelle.saulnier@univ-lyon1.fr (J.S.); florence.lagarde@isa-lyon.fr (F.L.); carole.chaix-bauvais@univ-lyon1.fr (C.C.); 2 Institute of Chemical Sciences, University of Rennes 1, 35000 Rennes, France; 3 University of Lyon, LAGEP, 69622 Villeurbanne, France; yves.chevalier@univ-lyon1.fr

**Keywords:** vesicles, polydiacetylene, biosensing, bacteria, toxins, virus, peptides

## Abstract

Polydiacetylene (PDA) inserted in films or in vesicles has received increasing attention due to its property to undergo a blue-to-red colorimetric transition along with a change from non-fluorescent to fluorescent upon application of various stimuli. In this review paper, the principle for the detection of various microorganisms (bacteria, directly detected or detected through the emitted toxins or through their DNA, and viruses) and of antibacterial and antiviral peptides based on these responsive PDA vesicles are detailed. The analytical performances obtained, when vesicles are in suspension or immobilized, are given and compared to those of the responsive vesicles mainly based on the vesicle encapsulation method. Many future challenges are then discussed.

## 1. Introduction

The demand for new sensing technologies that can serve as alerts for bacterial contamination has significantly increased in recent years because of incidents of food poisoning, bioterrorism alerts, and anthrax scares. Numerous technologies for bacterial detection have been developed [1]. Nevertheless, many methods employed for pathogen sensing provide results after relatively long time spans (several hours to days in the case of culture-based methods). Other currently employed technologies often involve complex detection mechanisms that require specialized instrumentation, trained personnel, and the need for complex sample preparation, which overall do not make possible uses in settings outside laboratory environments.

Polydiacetylene (PDA) has attracted significant scientific and technological interest in recent years because of its unique chromatic properties. Specifically, PDA has been shown to self-assemble into organized vesicles and films, forming an ene-yne conjugated framework that absorbs light in the visible region of the electromagnetic spectrum and consequently appears intensely blue [2]. Furthermore, it has been shown that external perturbations, primarily affecting the reorganization of the pendent polymer side-chains as a result of enhanced surface pressure, give rise to stress-induced structural transformations of the PDA backbone, resulting in dramatic blue-to-red transitions. PDA also exhibits interesting fluorescence properties: no fluorescence is emitted by the initially polymerized blue-phase PDA, whereas the red-phase PDA strongly fluoresces.

Synthetic vesicles or liposomes based on phospholipids mixed with polyacetylene have been extensively used for mimicking cell membranes [3]. For this purpose, the molecular system produced should retain, as much as possible, the physico-chemical properties of the actual cell membrane (such as lipid and protein organization and fluidity). The elaboration of biosensors for hemolytic bacteria is based on the detection of their emitted toxin that has the specific property of forming pores in the cell membrane. Screening of molecules with antibiotic properties is also based on the specific properties of these molecules to form pores in the cell membrane. This review paper reports the main recent works that present PDA vesicle-based assays, involving this phenomenon, for the detection of bacteria, bacterial toxins and antibiotic peptides. The direct detection of bacteria based on the specific interaction with antibody- and aptamer-functionalized PDA vesicles is also reported, and both principles of detection are compared in terms of selectivity and sensitivity. The direct detection of viruses based on the specific interaction with receptor-functionalized PDA vesicles is also reported. The analytical performance of PDA vesicle-based assays is moreover compared to that of other types of responsive vesicles, involving mainly the vesicle encapsulation method.

Reviews of synthetic vesicles are mainly focused on vesicle encapsulation methods that enhance the sensitivity of sandwich immunoassays [4,5]. This aspect will not be included herein.

## 2. Physicochemical Characteristics of PDA Vesicles

### 2.1. Structure and Synthesis of PDA Vesicles

**Figure 1:**
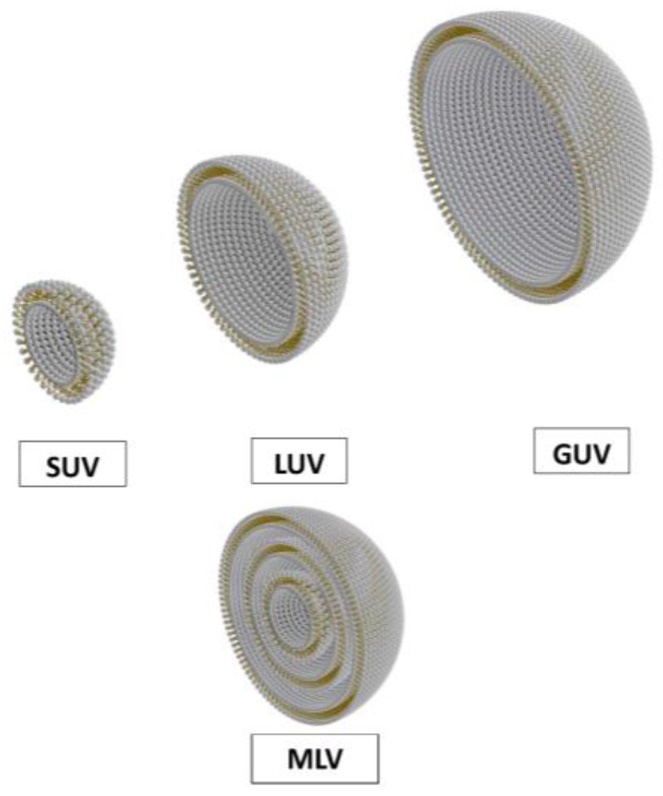
Different structures of vesicles: SUV (small unilamellar vesicles), LUV (large unilamellar vesicles), GUV (giant unilamellar vesicles), and MLV (multilamellar large vesicles). Vesicles are presented as hemivesicle to show the inside.

Synthetic or natural surfactants that can self-assemble as bilayers are the elementary molecules of vesicles or liposomes. The most common surfactants forming liposomes are phospholipids, the surface-active compound present in cell membranes; liposomes can then mimic biological membranes [3]. The structure of vesicles depends on the dispersion process [6]. The most common structures are multilamellar large vesicles (MLV), small unilamellar vesicles (SUV) of sub-micron diameter made of a single closed bilayer membrane, and giant unilamellar vesicles (GUV) of a few tens of micron in diameter (Figure 1).

The PDA vesicles are all unilamellar vesicles composed of one spherical mixed bilayer encapsulating probes or not. The general procedure for their preparation is summarized as follows: A mixture of phospholipids and diacetylenic acid is completely dissolved in chloroform [7], which is then evaporated. The dry lipid film is hydrated by the addition of an aqueous solution. The vesicle solutions are extruded through polycarbonate membranes or sonicated. Polymerization of PDA is then performed under UV light (254 nm).

The study of the influence of the UV doses on the stability of vesicles has shown that a higher degree of PDA polymerization improves their overall stability [8]. Moreover, the passive leakage of entrapped probes (i.e., fluorescent probes) is minimized when the degree of PDA polymerization is increased [8]. The composition of the lipid mixture can influence the biomimetic behavior of the obtained film, as demonstrated in Ref. [9].

Size-controlled fabrication of supramolecular vesicles using a microfluidic chip has been described [10]. The mean and standard deviation of the diameters of PDA vesicles produced by using the bulk method are respectively 88 and 31 nm and those of vesicles prepared with the microfluidic method are, respectively, 39 and 12 nm.

### 2.2. The Colorimetric Response of the PDA Vesicles and Formats of the PDA Vesicle-Based Assays

One of the more fascinating aspects of polydiacetylene chemistry is the color and chromism of the materials. The energy of electronic excitations, and therefore the color of the material, can be dependent upon many factors such as the original packing state of the monomers and the exposure of the polymeric material to environmental perturbations such as heat (thermochromism), mechanical stress (mechanochromism) or solvent (solvatochromism). The blue-to-red transition is associated with a conformational change of the PDA backbone from planar to non-planar and then more conjugated, the side chains being more ordered in the red phase. The color transitions of the polymerized vesicles are monitored by visible absorption spectroscopy: 620–640 nm (PDA blue form) and 490–540 nm (PDA red form) [2]. PDA red form also presents a fluorescence emission in the range 520–700 nm, when excited at 488 nm. As an example, colorimetric and fluorescent detection of melamine through PDA vesicles were compared. The intra/inter hydrogen bonding between melamine and the cyanuric acid receptor at the PDA vesicle surface induces perturbation of the PDA backbone and results in rapid and sensitive colorimetric/fluorescence change of the PDA vesicle. A detection limit of 1.0 ppm is obtained for colorimetric PDA liposome and 0.5 ppm for fluorescent PDA vesicle array [11].

The format of the colorimetric/fluorescence assay can be as that of a multiwell plate when vesicles are free in solution (see, for instance, Ref. [12]). To miniaturize the assay, patterned arrays are formed through the immobilization of vesicles on surfaces. Different surface modifications have been proposed for vesicle immobilization: aldehyde [13,14], amine [14,15] and α-cyclodextrin [16] functionalizations. An interlinker, ethylenediamine, which acts as a cross-linker between individual PDA vesicles, allows stabilization of PDA vesicles on a solid surface and the fluorescence signal is ten times higher than for the array without the interlinker [17].

### 2.3. The Physico-Chemical Characterization of the PDA Vesicles

Of the many physicochemical characterization methods used thus far, light scattering and microscopy methods provide the clearest information regarding the morphology of vesicles.

Dynamic light scattering (DLS) measurements allow the diffusion coefficient of the vesicles in the liquid suspension to be determined. This diffusion coefficient is converted into a mean diameter using the Stokes–Einstein relationship under the hypothesis that vesicles are spherical in shape. This assumption can be unsuitable when vesicles are flexible and their shape strongly fluctuates; this is the cases of large unilamellar vesicles. DLS does not allow the user to infer the shape or discriminate between unilamellar and multilamellar vesicles, nor does it allow detection of pores or holes through the lipid bilayer, nor discrimination between closed bilayer (vesicles) and fragments of bilayers (bicelles). DLS provides quite satisfactory data in the case of vesicles smaller than 1 µm. Nanoparticle tracking analysis (NTA), where the trajectories of individual scattering objects are observed under a microscope and their displacement is related to each object’s size, is considered the gold standard technique for PDA vesicle characterization. Both techniques are influenced by shape in the same manner.

**Figure 2:**
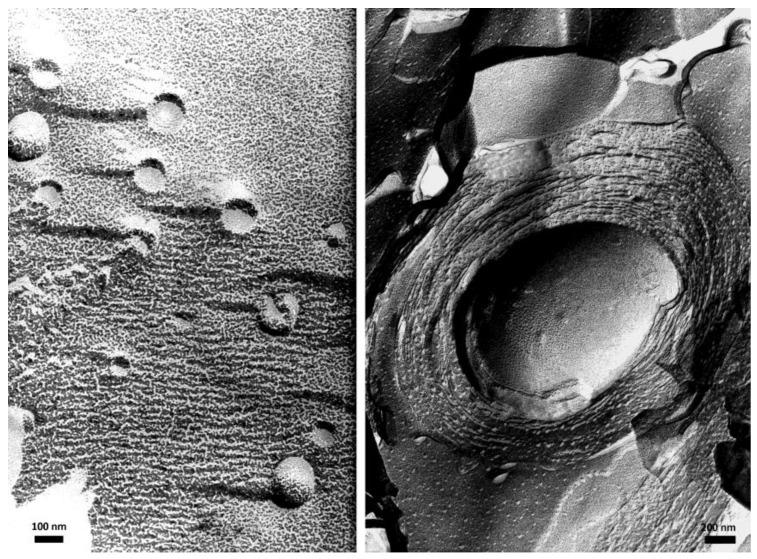
TEM images of large unilamellar vesicles (LUV) (**left**) and multilamellar giant vesicles (MGV) (**right**), made of bilayers of synthetic double chain zwitterionic surfactants, taken after preparation by freeze-fracture and replication of the fracture section. LUV appear as small circles being either full or having an empty water pool inside depending on whether the fracture propagated across the vesicles or along their external surface. MGV appear as onion-like stacks of lipid bilayers. Such concentric bilayers fill the whole vesicle; the empty hole in the middle corresponds to part of the vesicle center that has been detached when fracturing the frozen sample.

Transmission electron microscopy (TEM) provides high resolution pictures of the vesicles, allowing the discrimination of unilamellar and multilamellar vesicles, and possibly the thicknesses of the lipid bilayers and the water layers in between them (in the case of multilamellar vesicles). Classical TEM requires the samples to be dried before observation, so drying is not expected to change the organization, and the images should reveal the structure present in the aqueous suspension. It is often useful to enhance the contrast using heavy metal staining agents such as uranyl acetate. Again, it is hoped that staining will not disturb the structure. Either cryo-TEM or TEM of a replica prepared by the freeze-fracture technique allows more reliable observations of the structure prevailing in the liquid suspension. An example of such images of large unilamellar and multilamellar giant vesicles, made of bilayers of synthetic double chain zwitterionic surfactants [18], is given in Figure 2.

The colloidal stability of PDA vesicles requires strong enough repulsions between them to prevent coagulation. Quite strong electrostatic repulsions come from the presence of the anionic carboxylic groups as heads of PDA chains. Electrostatic effects can be assessed by electrophoretic measurements of the zeta potential. Since most charged species are salts of weak acids, it is wise to measure the zeta potential as a function of pH and determine the isoelectric point. Efficient electrostatic stabilization requires the pH to be shifted by at least one or two units from the isoelectric point.

The most interesting formulations of lipid components on the organization of lipid membranes could also be investigated by compression in a Langmuir trough. The pressure-area isotherms of mixed monolayers including the same lipid components are registered, giving information on the overall lipid compaction. The elasticity of a PDA mixed Langmuir film was studied in Ref. [19].

Atomic force microscopy [20] and Total Internal Reflection Fluorescence (TIRF) allows the shape and of the viscosity of the individual biomimetic vesicles to be evaluated. TIRF single vesicle measurements were performed to determine the vesicle rupture lag time in the presence of antiviral peptides [21]. It was demonstrated that C5A peptide presents a potent vesicle rupture activity, this activity being independent of vesicle diameter. AH peptide is highly membrane-active while it preserves vesicle size-selectivity. This point influences the range of enveloped viruses that is targeted.

## 3. Transducing Principles and Preparation of Responsive Biomimetic Vesicles

**Figure 3:**
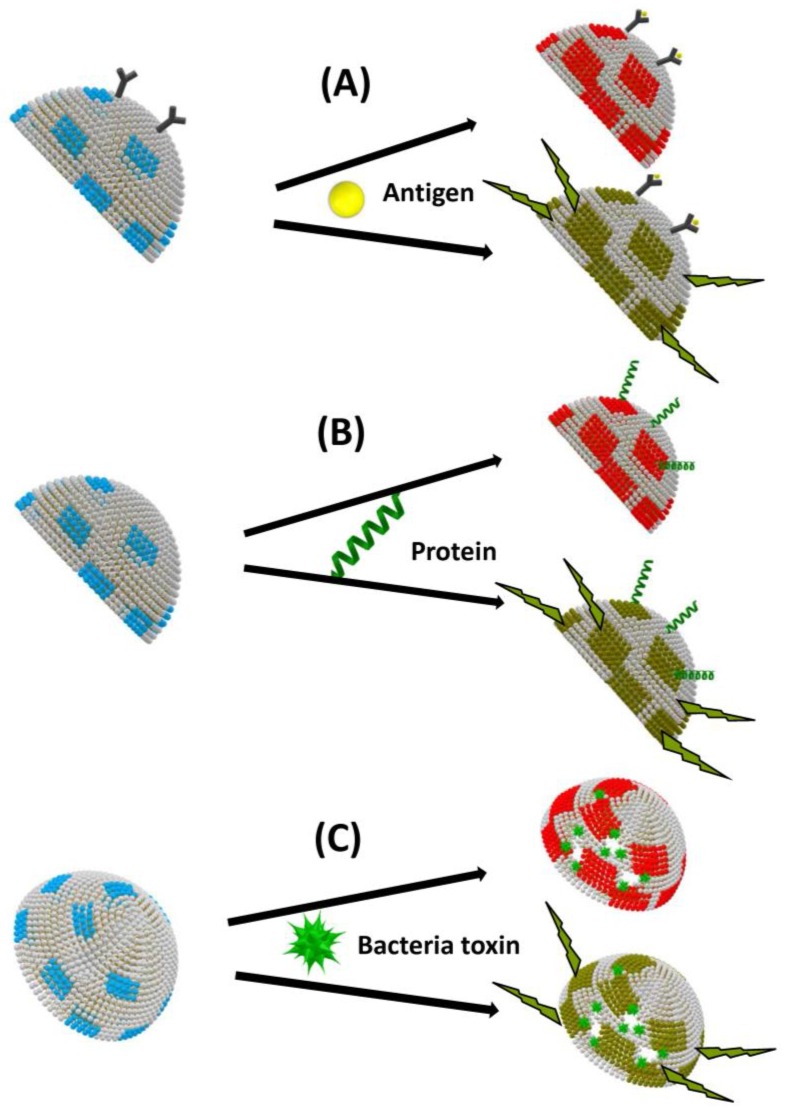
Colorimetric (upper line) and fluorescence (lower line) biosensing based on biomimetic vesicles comprising polydiacetylene (PDA) (blue and red parts) induced by external stimuli: (**A**) surface binding; (**B**) insertion; and (**C**) pore formation. Vesicles are presented as hemivesicles.

**Figure 4:**
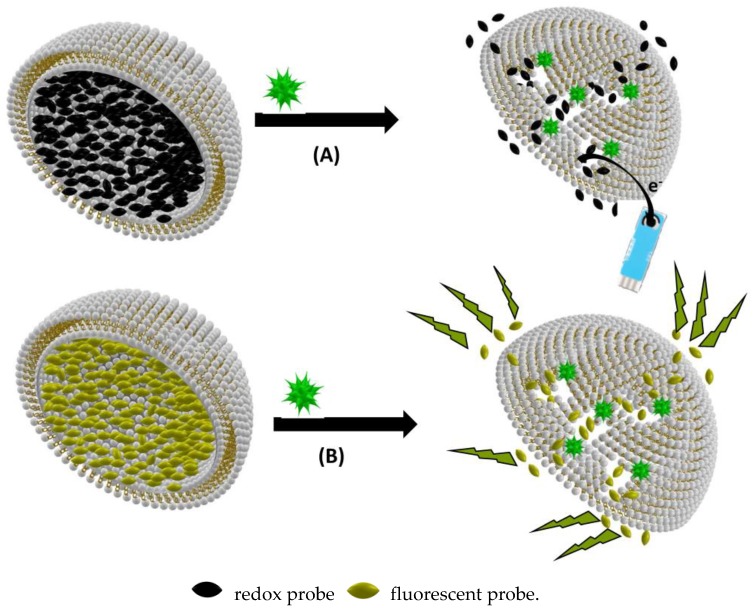
(**A**) Amperometric biosensing based on biomimetic vesicle encapsulation of redox probes; and (**B**) fluorimetric biosensing based on biomimetic vesicle encapsulation of fluorescent probes. Vesicles are presented as hemivesicles to show the inside.

Two main transducing principles are implemented in responsive biomimetic vesicles:

(1) Biomimetic PDA vesicles, PDA being used as a transducer for biological sensing, have been used as useful platforms for analysis and rapid screening of biomolecular recognition events [22]. Conjugated PDA is a remarkable polymeric system which exhibits unique organization and chromatic properties. This polymer has a strong blue color, due to electron delocalization within the conjugated double bonds, giving rise to absorption at around 650 nm in the visible region of the electromagnetic spectrum. Importantly, PDA can undergo both rapid blue–red color transitions (upper lines in Figure 3) and concomitant fluorescence transformations (lower lines in Figure 3), induced by external stimuli such as surface binding, insertion or pore formation, which disturb electron distribution along the polymer chains.
(2) Other types of responsive vesicles are based on the vesicle encapsulation method: fluorescent dye or redox species being encapsulated in the vesicle. These assays were mainly for the detection of species presenting pore-forming functions such as bacterial toxins or antibacterial substances (Figure 4).

An example of fluorescent vesicle-based biosensor for organophosphorous pesticides (OP) detection was developed by encapsulating the enzyme acetylcholine esterase and the pyranine fluorescent indicator in an egg phosphatidylcholine liposome. The enzyme substrate passes through porine channels and induces a decrease of pyranine fluorescence signal by decreasing the local pH. When the enzyme is incubated with OP, its activity decreases, inducing an increase of the fluorescence signal in the presence of the same concentration of substrate [23]. Another fluorescent liposome based system contains specific pyrenyl amphiphiles: upon the interaction with the target enzyme, thymidine phosphorylase, the variation of excimer/monomer ratio allows its presence to be specifically detected [24].

## 4. PDA Vesicle-Based Assays for Bacteria Detection

### 4.1. Direct Detection of Bacteria

The published papers dealing with the direct detection of bacteria that use mixed PDA vesicles are based on optical detection: colorimetric detection due to blue–red transition of PDA under mechanical stress or fluorescence detection in the presence of a fluorophore grafted on a diacetylenic acid chain (Table 1). The interaction with the bacteria membrane can be ensured by the following specific molecules

(1) either inserted in the bilayer membrane: long chain glucoside [25,26,28] and sphingomyelin [31,32]
(2) or grafted on the diacetylenic acid chain: antibody [28] and aptamer [30].

**Figure 5:**
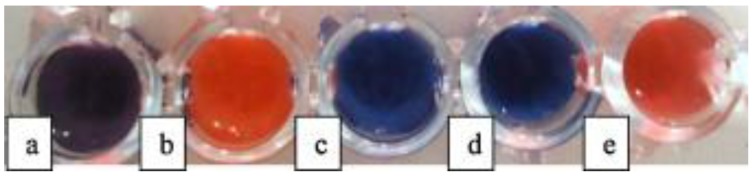
PCDA/SPH/Cholesterol/Lysine vesicles added to TSB (0.1%) and aqueous saline at pH 6.0 with: (**a**) *E. coli*; (**b**) *P. aeruginosa*; (**c**) *S. aureus*; (**d**) *L. plantarum*; and (**e**) *S. Choleraesuis* (10^8^ CFU/mL). Reprinted with permission from [31]. Copyright 2017 Elsevier.

The colorimetric detection of ligand/receptor interactions through physical incorporation of receptors within lipid/PDA vesicles offers important advantages over chemical attachment of recognition units to the PDA itself. First, the chemical derivatization of PDA can be technically demanding and the organic synthesis procedures limit the scope of this approach. Furthermore, attaching additional chemical units onto the diacetylene monomers often disrupts the organization and the self-assembly of the monomers and hence compromises polymerization. Consequently, the abundance of recognition modules in derivatized PDA vesicles is low. Such limitations are generally not encountered when the recognition element is incorporated in the lipid/PDA bilayer. This point is demonstrated when comparing the obtained detection limit using PCDA (10,12-pentacosadyinoic acid) functionalized with a LPS (lipopolysaccharide) aptamer (10^4^ CFU/mL of *E. coli*) [30] and the obtained detection limit with SPH (sphingomyelin) incorporated in a polyacetylene vesicle (1–10 CFU/mL of *S. choleraesuis*) [32]. The latter PDA vesicle-based assay was also tested for other types of bacteria in a high concentration of bacteria: 10^8^ CFU/mL. In Figure 5, it appears that *P. aeruginosa* was also detected [31]. The selectivity of detection is closely dependent on the recognition molecule as well as the solution conditions (pH value) [31,32].

Very rapid tests for the detection of bacteria in real samples were then designed using these mixed PDA vesicles for the detection of *Salmonella choleraesuis* in chicken meat [32].

**Figure 6:**
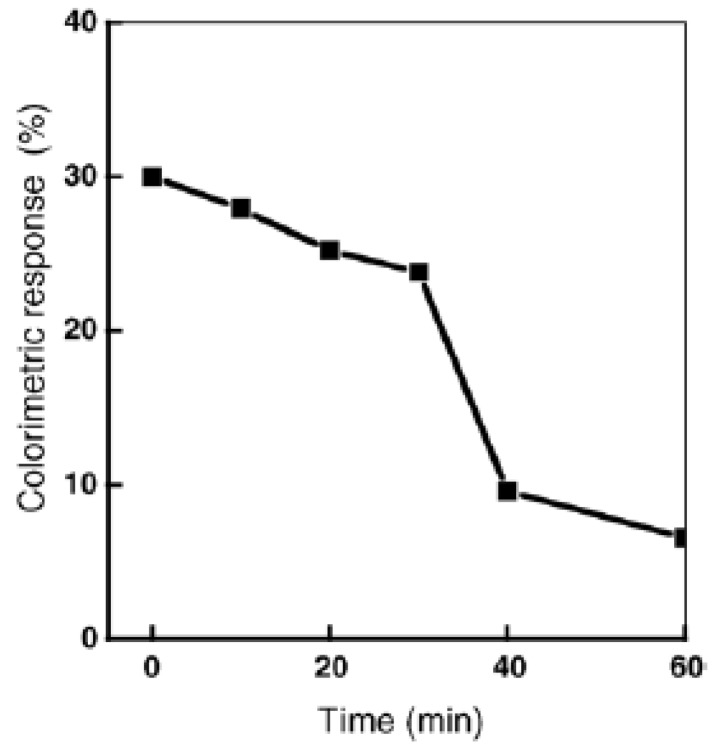
Colorimetric transition of mixed polyacetylene vesicles as a function of light irradiation time in the presence of TiO_2_ in *E. coli* K12 suspension. Reprinted with permission from [33]. Copyright 2005 Elsevier.

Real time monitoring of the photocatalytic sterilization process in the presence of TiO_2_ colloid was obtained by recording the colorimetric response (blue–red transition) of mixed polydiacetylene vesicles in the presence of *E. coli* (Figure 6) [33].

Bacterial RNA was detected by fluorescence measurements through conjugation with on-chip immobilized PDA vesicles, previously grafted with complementary DNA probes. Different types of crude cell lysate (*E*. *coli*, *L. monocytogenes*, and *S*. *enteritidis*) were incubated together with the specifically grafted vesicles. When target bacteria were matched with DNA probes, increased fluorescence intensities were observed. Although slight fluorescence, corresponding to non-specific signal, is detected, the level of fluorescence is much lower than that of matched probes. The detection limit was determined as 10^4^–10^5^ CFU/mL [16].

### 4.2. Indirect Detection of Hemolytic Bacteria through Toxin Detection

Pathogenic bacteria produce a large variety of toxins and virulence factors. Hemolytic bacteria are pathogenic bacteria that produce pore-forming toxins, ultimately resulting in cell death by necrosis or apoptosis [34]. PDA liposomes have been synthesized to detect this type of toxins through electrochemical or optical methods (colorimetry or fluorimetry) (Table 2). To mimic the cell membrane, the mixed bilayer is composed of a mixture of phosphocholine (1,2-dipalmitoyl-*sn*-glycero-3-phosphocholine (DPPC), 1,2-bis(10,12-tricosadiynoyl)*sn*-glycero-3-phosphocholine (PC-DIYNE), and 1,2-dimysristoyl-*sn*-glycero-3-phosphosphocholine (DMPC)), mixed with diacetylene monomers (glycine-terminated diacetylene monomer (GLY-PDA), 10,12-tricosadiynoic acid (TCDA), *N*-(10,12-pentacosadiunoyl)-glycine (Gly-PCDA), and 1,2-bis(10,12-tricosadiynoyl)*sn*-glycero-3-phosphocholine (PC-DIYNE)) and cholesterol as a bait molecule, since the first step for pore formation is believed to be toxin binding to cholesterol. For electrochemical detection, redox compounds such as ferrocene, hexacyanoferrate or 2,6-dichlorophenolindophenol, are entrapped in the vesicles [35,36,37,38] or inserted in the bilayer membrane [36,37]. Using ferrocene-PDA based vesicles, when the toxin is trapped by the receptor (ganglioside GM1), the toxin-receptor complex blocks the charge transfer route of the ferrocene probes to the electrode surface [36].

The optical techniques are based on the direct colour change of PDA through pore formation [39,40,41,43,46] or on the detection of released dye previously encapsulated in the vesicles [42,44,45].

Different types of toxins were detected such as steptolysin O from *S. pyrogenes* and rhamsolipid from *P. aeruginosa*.

For streptolysin O, detected using amperometry via the redox probe (hexacyanoferrate) release, the obtained detection limit is 5 HU (hemolytic unit) [35] while when detected by colorimetry through the blue–red transition, the obtained detection limit is 20 HU [39], showing that the method of vesicle encapsulation leads to lower detection limits.

**Figure 7:**
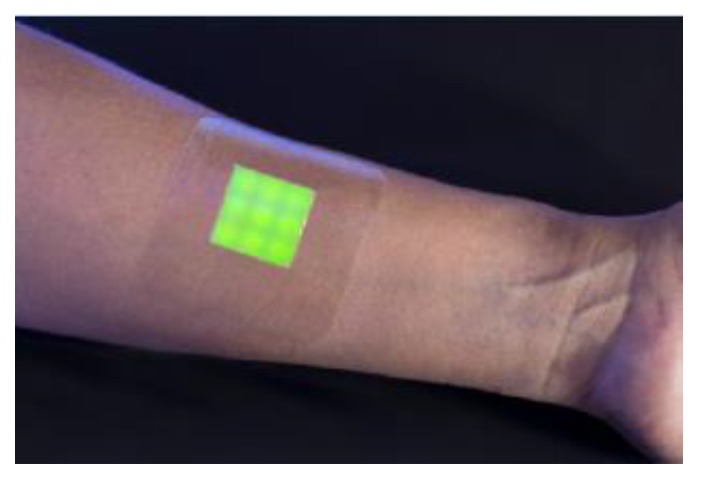
An intelligent hydrogel wound dressing based on fluorescent dye release from polydiacetylene vesicles. Reprinted with permission from [47]. Copyright 2016 American Chemical Society.

An intelligent hydrogel wound dressing has been made of a hydrated agarose film in which the fluorescent dye-containing vesicles were mixed with agarose and dispersed within the hydrogel matrix. The dressing indicated a clear fluorescent/color response within 4 h, only observed when in contact with biofilms in the wound, produced by a pathogenic strain. (Figure 7) [47].

### 4.3. Screening of Molecules with Antibiotic Properties

Assays providing rapid, easy evaluation of the interactions between antimicrobial substances and PDA bilayer-based vesicles as the cell membrane model could significantly improve screening of substances with effective microbial properties, as well as contribute to the elucidation of their structural and functional properties.

Due to their optical properties, polydiacetylene based vesicles were generally used for the design of these assays (Table 3). The first proof of the concept was demonstrated for different antimicrobial peptides in Ref. [48]. The composition of the mixed bilayer was optimized to improve the blue–red transition in terms of intensity and response time. Lipopolysaccharide (LPS) was inserted to promote the interaction with antibacterial peptide [50,51]. Lipid extracts from the red alga *Porphyridium cruentum* strain 1380.1/PDA vesicles were tested for the colorimetric detection of melittin and of polymixin B [52]. When these lipids present a lower total number of double bonds in the acyl residues, these antibacterial peptides induce higher colorimetric response, which might be due to higher fluidity within the lipid bilayer. Increased rigidity of the lipid moieties is expected to reduce penetration of the antibacterial peptide into the lipid bilayers, then resulting in peptide binding at the lipid headgroup region within the lipid/PDA vesicles. Such surface interaction is expected to induce greater perturbation of the pendant polymer side chains within the PDA matrix. 

Phospholipid vesicles inserting highly fluorescent dye allowed the very sensitive detection of alamethicin, an antibiotic peptide [49].

**Figure 8:**
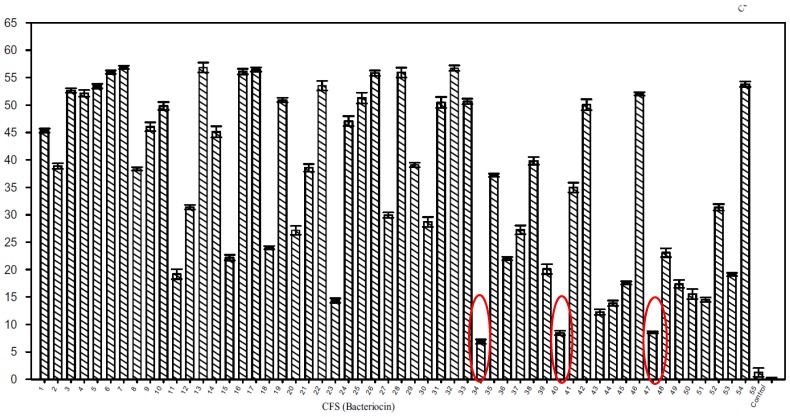
Color change of DMPE/TRCDA vesicles treated with 50 µL cell-free supernatant of 54 lactic acid bacteria strains. Reprinted with permission from [12]. Copyright 2017 Springer Nature.

The antimicrobial properties of metabolites of soil microfungi were tested through a colorimetric assay using PDA-based vesicles [53]. This assay was also applied to the high throughput screening of peptides—specifically bacteriocins—produced by lactic acid bacteria [12]. Figure 8 presents the percentage of color change of 1,2-dimysristoyl-*sn*-glycero-3-phosphoethanolamine (DMPE)/10,12-tricosadiynoic acid (TRCDA) vesicles treated with 50 µL cell-free supernatant of 54 lactic acid bacteria strains.

It appears that for lactic acid bacteria strains LV39 (well 34), LB52 (well 40) and LB64 (well 47) the colour change is weak, compared to that of the other strains. These strains were considered to be bacteriocin non-producers and some of the other strains with a colorimetric response higher than 50%, as bacteriocin producers.

PDA based liposome arrays for antibiotic detection, with PIP2 phospholipids as neomycin receptors, were schematized [54].

## 5. PDA Vesicle Based Assays for Detection of Influenza Viruses 

PDA vesicle-based assays were developed mainly for the detection of influenza viruses (cf Table 4). Influenza virus particles are enveloped by a lipid bilayer to which the hemagglutinin (HA) lectin is anchored. HA binds to terminal a-glycosides of sialic acid on cell-surface glycoproteins and glycolipids, initiating cell infection by the virus. The same type of interaction was then biomimicked on the PDA vesicle surface. The first proof of concept of the direct detection of influenza viruses through a red–blue transition of sialic acid bound to functionalized PDA vesicles was described in 1995 [55]; a detection limit of 11 × 10^7^ virus particles was obtained through colorimetric measurements. The same strategy was also proposed in Ref. [56,57,58]: sialic acid was grafted on β-glucoside and sialic and lactose moieties were grafted on a glucoside chain for insertion in the PDA layer. Different other types of virus receptor were grafted onto PDA moieties: antibodies [59,60] and peptides [61]. 

## 6. Conclusions and Future Directions

This review presents the state-of-the-art of mixed PDA based vesicles formulated to mimic cell membranes and how they constitute actual nanosensors for the direct detection of bacteria or viruses, of bacterial toxins (bacterial virulence) and of antibacterial and antiviral peptides, through direct blue–red transition or through the passive release of encapsulated probes. 

The colorimetric assays based on these PDA based vesicles are very cheap and easy to handle. They have been applied to the high throughput screening of toxins of natural origin (fungi [53] and bacteria [12]) through the design of a biosensing platform [12]. They have also been applied to rapid bacterial detection in food [32,40]. It has been observed that the vesicle encapsulation method, leading to an amplification effect, provides a lower detection limit, through the electrochemical detection of redox probes or the optical detection of fluorescent probes. 

However, there are still important bottlenecks that limit the development of PDA vesicle-based bioassays. In aqueous solution, the sensitivity of PDA vesicles is usually unsatisfactory for the main applications in medical diagnostic and food safety. To increase the color change, a high concentration of analyte is required. The reversibility of PDA vesicle-based bioassays is also attractive to obtain a reusable sensor. Reversible responses can only be achieved by heating, pH change or UV light and no PDA sensors for biochemical analytes are actually reversible. Rigorous theoretical studies and simulations of the important transitions, leading to the color change are required to develop a clearer understanding of their origin. Improvement of stability under various conditions is a major concern, particularly rehydration efficiency to enable dry sensor forms to be developed that would be a necessary step towards the potential commercialization of point of care systems based on microarrays of PDA vesicles. Some cryoprotectants could be used for this purpose [62].

The ultimate solution to all these issues will enable the development of highly sensitive PDA vesicle-based biochips for the commercialization of convenient rapid tests, such as points-of-care for medical diagnostics, intelligent wound dressing or colorimetric tests for food safety.

## Figures and Tables

**Table 1:** PDA vesicles for detection of bacteria.

Composition of the Bilayer	Diameter of Vesicles (µm)	Type of Transduction	Type of Bacteria	Bacteria LOD (CFU/mL)	Reference
TCDA/2% DGG		Colorimetry	*E. coli (ATCC25922)*	Not given	[25]
HCDA/DL3		Colorimetry	*E. coli*	10^8^	[26]
PCDA-ABA/PCDA-biotin-streptavidin-anti *E. coli* antibody/(20–30%) DMPC		Fluorescence	*E. coli*	1.2 × 10^7^	[27]
PCDA/glucose-tagged lipid or glucose-PCDA/rhodamine tagged DMPC	15–60 (GUVs)	Colorimetry Fluorescence (FRET)	*E. coli*	3.3 × 10^5^	[28]
TRCDA/DMPC		Colorimetry	*E. coli*	10^8^ (drinking water)	[29]
PCDA vesicles functionalized with LPS binding aptamer		Colorimetry	*E. coli (O157:H7)*	10^4^	[30]
PCDA/SPH/cholesterol/Lysine Lysine concentration 6.7 µg/mL, pH 6.5	0.2	Colorimetry	*S. choleraesuis*	10^8^	[31]
PCDA/SPH/cholesterol/Lysine Lysine concentration 6.7 µg/mL, pH 6.0	0.2		*S. choleraesuis*	10^0^–10^1^ in chicken meat	[32]

DGG: dioctadecyl glycerylether-β-glucoside; DL3: 3,6,9,12-tetraoxa-10-cholest-2-acetamido-2-desoxy-β-d-glucopyranoside; DMPC: 1,2-dimysristoyl-*sn*-glycero-3-phosphatidylcholine; GUVs: giant unilamellar vesicles; HCDA: 2,4-heneicosadiynoic acid; LOD: limit of detection; LPS: lipopolysaccharide; MLVs: multilamellar vesicles; PCDA: 10,12-pentacosadyinoic acid; PCDA-ABA: 10,12-pentacosadyinoic acid grafted with abscisic acid; PCDA-biotin: 10,12-pentacosadyinoic acid grafted with biotin; SPH: sphingomyelin; TCDA: tricosa-2,4-diynoic acid; TRCDA: 10,12-tricosadiynoic acid.

**Table 2:** PDA vesicles for detection of hemolytic bacteria and toxins.

Composition of the Bilayer	Nature of the Encapsulated Probe	Type of Transduction	Type of Hemolytic Bacteria	Type of Toxin	Toxin LOD nM	Bacteria LOD (CFU/mL)	Reference
**Electrochemical detection**							
GLY-PDA/Fc-PDA/receptor ganglioside GM1		Amperometry	*E. coli*	*E. coli* Heat-labile enterotoxin	36		[36]
Phosphatidylcholine/cholesterol/ diacetyl phosphate/1-octadecanethiol	hexacyanoferrate	Amperometry	*S. pyrogenes* A and C	Streptolysin O	0.025 5 HU *		[35]
phosphatidylcholine	2,6-dichlorophenol/indophenol	Amperometry	*L. monocytogenes* NCTC 7973	__	__	5 × 10^6^	[37]
Phosphatidylcholine/2,6-dichlorophenolindophenol		Amperometry	*L. monocytogenes* NCTC 7973	__	__	5 × 10^5^	[37]
DPPC/cholesterol/TCDA	hexacyanoferrate	Amperometry	*P. aeruginosa* PAO1 *S. aureus* USA300	Rhamnolipid Delta toxin	11,000 29,000		[38]
**Optical detection**							
Gly-PCDA/PC-DIYNE/cholesterol		Colorimetry	*S. pyrogenes* A and C	Streptolysin O	0.10 20 HU *		[39]
DMPC/10,12-tricosadiynoic acid		Colorimetric	*Salmonella enterica*	Bacterial supernatant		10^9^ bacteria	[40]
Glycopolydiacetylene		Colorimetry	*E. coli* O157:H7	Shiga toxin		1.2 × 10^6^	[41]
DMPE /DMPE /TCDA/cholesterol	carboxyfluorescein	Fluorescence	*P. aeruginosa* PAO1 *S. aureus MSSA* 476			10^4^	[42]
PCDA/TDER		Colorimetry	*S. aureus* (ATCC 6538) *E. coli* (ATCC 11229)	Bacterial supernatant		10^8^ (spiked apple juice)	[43]
DPPC /cholesterol/TCDA /DPPE	carboxyfluorescein	Fluorescence	*P. aeruginosa*	Rhamnolipid	40,000	10^6^ CFU/mL	[44]
Hyaluronic acid/caprolactone	7-amino-4-methylcoumarin	Fluorescence	*S. aureus*	hyaluronidase	47 U/mL		[45]
Amine terminated PDA		Colorimetry Fluorescence	*B. subtilis*, *P. aeruginosa*	surfactin	16,500	1.8 × 10^3^	[46]

DMPC: 1,2-dimysristoyl-*sn*-glycero-3-phosphosphocholine; DMPE: 1,2-dimysristoyl-*sn*-glycero-3-phosphoethanolamine; DPPC: 1,2-dipalmitoyl-*sn*-glycero-3-phosphocholine; DPPE: 1,2-dipalmitoyl-*sn*-glycero-3-phosphoethanolamine; Fc-PDA: *N*-(10,12-pentacosadiunoyl)acetylferrocene; Gly-PDA: glycine-terminated diacetylene monomer; GLY-PCDA: N-(10,12-pentacosadiunoyl)-glycine; GM1: ganglioside; LOD: limit of detection; PCDA: 10,12-pentacosadyinoic acid; PC-DIYNE: 1,2-bis(10,12-tricosadiynoyl)-*sn*-glycero-3-phosphocholine; TCDA: 10,12-tricosadiynoic acid; TDER: N-[(2-tetradecanamide)-ethyl]-ribonamide; * HU (hemolytic unit) is defined as the amount of protein that causes 50% lysis of a 2% red blood cell suspension in PBS at pH 4.

**Table 3:** PDA vesicles for detection of antibacterial peptides.

Composition of the Bilayer	Encapsulated Probe	Type of Transduction	Nature of Antibiotic	Antibiotic LOD (µM)	Reference
DMPC/PCDA	No	Colorimetry	K7L-melittin Magainin II	100	[48]
POPC	Dipicolinic acid/Tb^3+^	Fluorescence	alamethicin	0.25	[49]
LPS/DMPC/PCDA	No	Colorimetry	Indolicidin analog (proline replaced by alanine)	30	[50]
LPS/DMPC/PDA	No	Colorimetry	Polymixin B derivatives	3	[51]
Lipid extracts from the red algae *Porphyridium cruentum* strain 1380.1/PCDA	No	Colorimetric	Melittin Polymixin B	1 1	[52]
DMPC/TCDA	No	Colorimetry	Antimicrobial membrane-active metabolites of soil fungi (strain 08-29-2)		[53]
DMPE/TRCDA	No	Colorimetry	Nisin Antibacterial peptides		[12]

DMPC: 1,2-dimyristoylphosphatidylcholine; LOD: limit of detection; LPS: lipopolysaccharides; PCDA: PCDA: 10,12-pentacosadyinoic acid; POPC: palmitoyl oleoyl phosphatidylcholine; TCDA, TRCDA: 10,12-tricosadiynoic acid.

**Table 4:** PDA vesicles for detection of viruses.

Composition of the Bilayer	Diameter of Vesicles	Type of Transduction	Type of Virus	Virus LOD	Reference
95% PCDA/5% sialic acid derivatized PCDA		colorimetry	Influenza	11 × 10^7^ virus particles	[55]
95% PCDA/5% sialic acid derivatized PCDA		colorimetry	Influenza	8 × 10^7^ virus particles	[56]
PCDA/5% S-sialo PCDA		colorimetry	Influenza X-31	0.78 HAU	[57]
PDMA/DMPC/G1/G2 G1: sialic acid-β-glucoside G2: lactose-β-glucoside	10–20 nm	colorimetry	H5N1 Avian influenza	10 ng/mL hemagglutinin	[58]
PCDA Anti-H5N1 monoclonal antibody grafted on vesicle surface	117 nm	colorimetry fluorescence	H5N1 Avian influenza	30 ng/mL HAQ 1 ng/mL HAQ	[59]
PCDA/DMPC Anti-HA monoclonal antibody grafted on vesicle surface		colorimetry	H5 influenza	0.53 copies/µL	[60]
PEP-PCDA	~50 nm	colorimetry	H1N1 influenza	10^5^ PFU	[61]

DMPC: dimyristoylphosphatidylcholine; HAQ: target antigen of H5N1 Avian influenza virus strain; LOD: limit of detection; PCDA: 10,12-pentacosadiynoic acid; PEP-PCDA: peptide-functionalized 10,12-pentacosadiynoic acid.
